# Defect-Induced Transport Enhancement in Carbon–Boron Nitride–Carbon Heteronanotube Junctions

**DOI:** 10.1021/acs.jpclett.3c00004

**Published:** 2023-02-16

**Authors:** Laith A. Algharagholy, V. M. García-Suárez

**Affiliations:** † Department of Physics, College of Science, University of Sumer, Al Rifaee, 64005, Thi-Qar, Iraq; ‡ Departamento de Física, Universidad de Oviedo & CINN, Oviedo, 33007, Spain

## Abstract

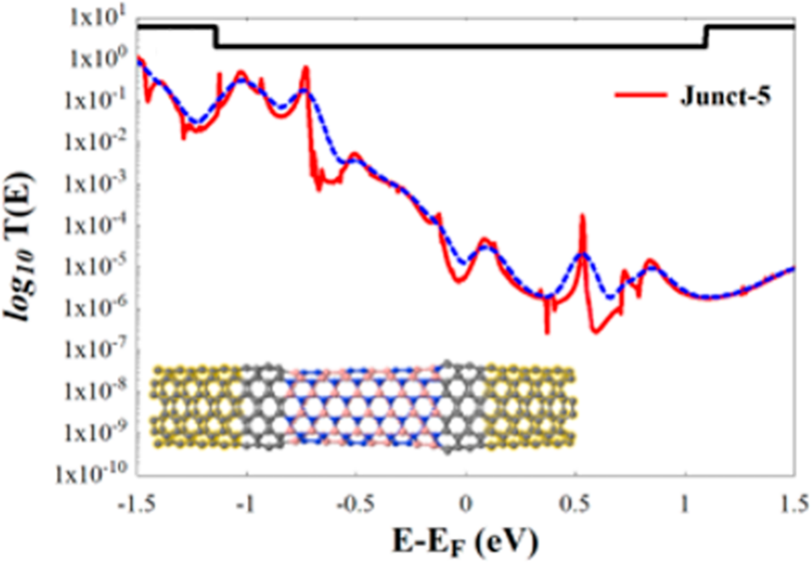

New heteromaterials, particularly those involving nanoscale elements such as nanotubes, have opened a wide window for the next generation of materials and devices. Here, we perform density functional theory (DFT) simulations combined with a Green’s function (GF) scattering approach to investigate the electronic transport properties of defective heteronanotube junctions (hNTJs) made of (6,6) carbon nanotubes (CNT) with a boron nitride nanotube (BNNT) as scatterer. We used the sculpturene method to form different heteronanotube junctions with various types of defects in the boron nitride part. Our results show that the defects and the curvature induced by them have a nontrivial impact on the transport properties and, interestingly, lead to an increase of the conductance of the heteronanotube junctions compared to the free-defect junction. We also show that narrowing the BNNTs region leads to a large decrease of the conductance, an effect that is opposite to that of the defects.

Since their discovery by Sumio Iijima in 1991,^1^ carbon nanotubes (CNTs) have received tremendous research attention for their unique electronic,^2−5^ mechanical,^5−7^ optical,^8,9^ and thermal^10−12^ properties. Carbon nanotubes have different electronic behaviors; they can be semiconductors or conductors depending on the chirality, which is often difficult to control in synthesis.^13,14^ Boron nitride nanotubes (BNNTs), on the other hand, which were first discovered in 1995,^15^ exhibit a wide energy gap of approximately of 5 eV.^15,16^ A variety of techniques can be used for manufacturing CNTs and BNNTs, such as arc discharge,^13,17−19^ laser ablation,^20−22^ and chemical vapor deposition (CVD),^23−25^ which usually produce again a mixture of chiralities. Various research works have been undertaken on BNNT, which mainly focused on properties such as the electronic structure,^15,26,27^ and optical,^28−30^ thermal,^31,32^ and mechanical^26,30,33^ properties. Regarding the electronic structure, introducing defects in nanotubes has a nontrivial influence on their electronic structure,^34^ e.g., defect-induced deformations in CNTs^35−38^ lead to an extreme decrease in the electronic transport, whereas in BNNTs, defects^39−41^ produce a narrow energy gap. The most common defects in nanotubes are five–seven (Stone–Wales) defects.^38,42−45^ Previous studies including molecular dynamics (MD) calculations^46−50^ reported that such defects are stable even at high temperatures. On the other hand, modulating the periodic structure of pristine CNTs by other materials such as nitrogen and boron to form heteronanotubes, can modify their electronic properties,^51−54^ turning, for instance, a metallic CNT into a semiconductor.^52,54−57^

A variety of experimental techniques have confirmed the synthesis and characterization of nitrogen/boron-doped^58−63^ CNTs. Heterojunctions, such as carbon–boron nitride heteronanotube junctions are also energetically stable^52,64,65^ and have a conductance that can be tuned by altering the width of the boron nitride strips in the heteronanotube.^66^ Despite many difficulties involved in the preparation of carbon–boron nitride heterojunctions, experimental efforts have successfully synthesized them using different methods.^67−72^ In this work, we investigate the electronic transport properties of ideal heterojunctions consisting of (6,6) CNTs as left/right leads and a (6,6) BNNT as scattering region. We also consider the impact of different factors such as defects and the width of the scatterer (BNNT) on the transport properties.

To form the heteronanotube junctions (hNTJs) shown in Figure 1(b–g), we used the sculpturene method,^64^ which provides a methodology for creating deterministic carbon nanotubes (CNTs) through the reconstruction of bilayered graphene nanoribbons (bi-GNRs), including bilayered heteronanoribbons materials, such as graphene (G) and boron nitride (BN). Notice that, even though the strain can be large and act as a destabilizing factor in the initial stages of the formation of the nanotubes, the structures naturally evolve to such configurations after several relaxation steps, provided the initial ribbons are close enough to each other. This procedure opens a wide window of options for constructing topologically unique nanostructures. In this work, we started with AA-staked zigzag bilayered heteronanoribbons (bi-hNRs), each layer composed of graphene-boron nitride-graphene (G-BN-G), to form hNTJs, which consist of (6,6) CNTs as left/right leads and a boron nitride nanotube (BNNT) as scatterer (CNT-BNNT-CNT). To optimize the initial supercells (AA-staked zigzag bi-hNRs), we employed the SIESTA^73^ implementation of density functional theory (DFT). We used the local density approximation (LDA)^74^ with the Ceperley–Alder (CA) exchange correlation functional, and double-ζ polarized (DZP) basis sets of pseudoatomic orbitals, together with norm-conserving pseudopotentials. Notice that the LDA usually overestimates the binding, giving distances between atoms a bit smaller than the real ones, but works properly in general to describe van der Waals interactions^75^ such as those present in the initial stages of the design of the sculpturenes. The initial supercells were relaxed until all the forces were smaller than 0.005 eV/Å. To avoid the interaction between neighboring heterostructures, a vacuum space of 50 Å was included along the *X* and *Y* directions, whereas along the *Z* direction the structures were periodic. For the leads calculations, a *k*-point grid of 1 × 1 × 30 in the Brillouin zone was used. Once the final hNTJs were achieved, we took the mean field Hamiltonian (MFH) and overlap matrices from SIESTA, and, using the GOLLUM^76^ implementation of equilibrium transport theory, we calculated the low bias electron transmission coefficients *T*(*E*) and currents *I*.

**Figure 1:**
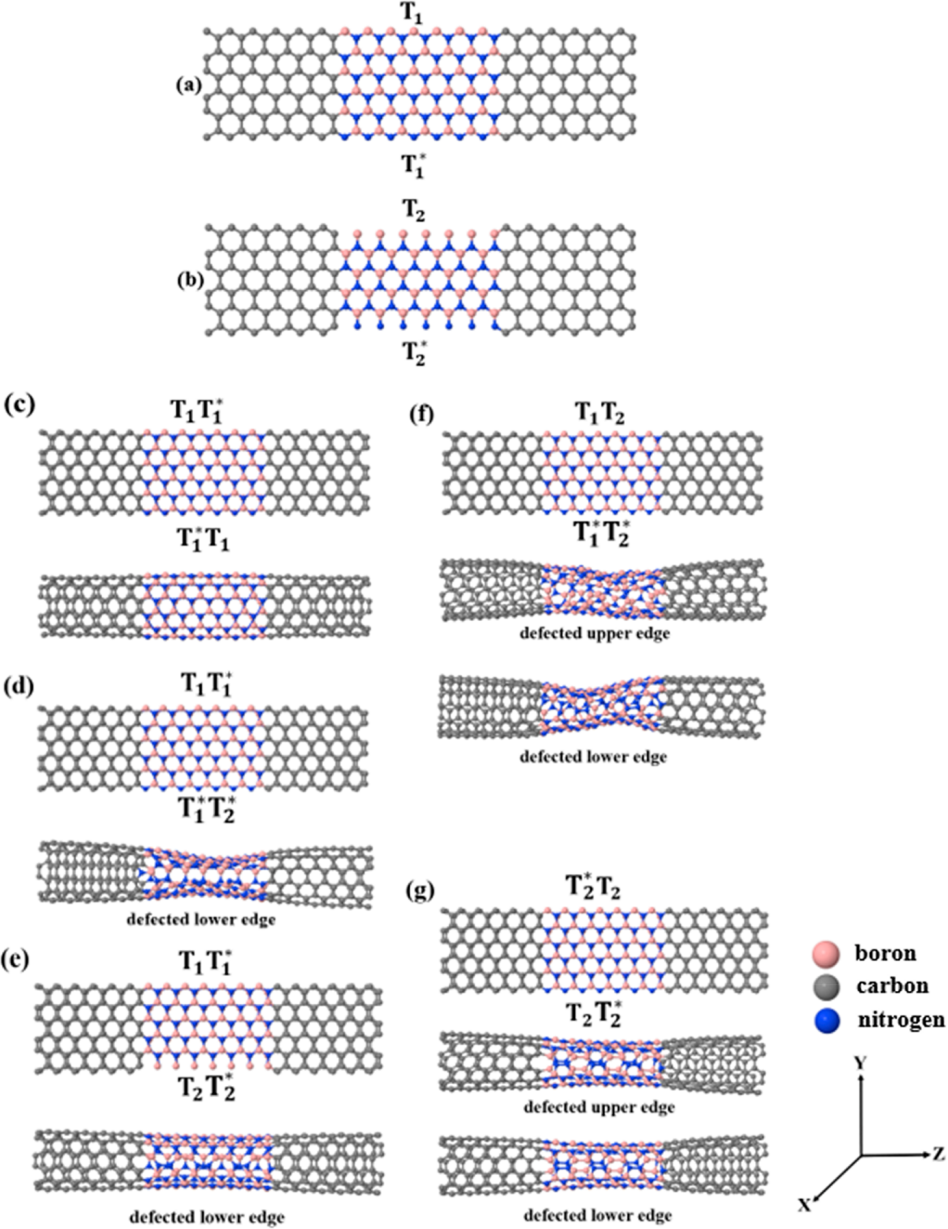
Single-layered zigzag hNRs with (a) T_1_ termination and (b) T_2_ termination. The top subfigures in figures (c–g) show the initial supercell obtained by cutting a zigzag AA-stacked bilayered hNRs, while the bottom subfigures show the relaxed sculpturenes, which make the CNT-BNNT-CNT heteronanotube junctions. Each figure corresponds to a different configuration of terminations: (c) with T_1_T_1_^*^ (top upper/lower edges) and T_1_^*^T_1_ termination (bottom upper/lower edges), (d) with T_1_T_1_^*^ termination (top upper edge with T_1_ termination and top lower edge with T_1_^*^ termination), and T_1_^*^T_2_^*^ termination (bottom upper and lower edges terminated with T_1_^*^ and T_2_^*^, respectively), (e) with T_1_T_1_^*^ termination (top upper edge with T_1_ termination and lower edge with T_1_^*^ termination) and T_2_T_2_^*^ termination (bottom upper edge with T_2_ and lower edges with T_2_^*^), (f) with T_1_T_2_ termination (top upper/lower edges are terminated with T_1_ and T_2_ terminations respectively) and T_1_^*^T_2_^*^ termination (top upper edge is with T_1_^*^ termination and bottom lower edge is with T_2_^*^ termination), and (g) with T_2_^*^T_2_ termination (top upper edge with T_1_^*^ termination and bottom lower edge with T_2_^*^ termination) and T_2_T_2_^*^ termination (top upper edge terminated with T_2_ termination and bottom lower edge terminated with T_2_^*^ termination).

For single-layered zigzag hNRs, there are four possible side edges of the BN region, as shown in Figure 1(a,b). We name these terminations as T_1_, T_1_^*^, T_2_, and T_2_^*^. In an ideal case, the upper/lower edges in the boron nitride region would have the T_1_ and T_1_^*^ configurations (Figure 1a). The T_2_ termination is created by eliminating a complete row of nitrogen atoms, while the T_2_^*^ termination is obtained after removing a complete row of boron atoms. Note that in the presence of T_2_ and T_2_^*^ terminations, shown in Figure 1b, the resulting hNTJs will have a defected BNNT region or scatterer (see below).

We conveniently refer from now on to the hNTJs shown in Figure 1(c–g) as Junct-1, Junct-2, Junct-3, Junct-4, and Junct-5, respectively. For more clarity, Figure S1 (Supporting Information) schematically shows the defects created in the relaxed BNNT (scatterer) of the hNTJs displayed in Figure 1(d–g). From Figure S1, we can see that a variety of defects with different geometries arise in the resulting hNTJs (Junct-2, Junct-3, Junct-4, and Junct-5) when the sculpturenes are made. Junct-2 and Junct-3 are relaxed with one defected side (bottom), while Junct-4 and Junct-5 are relaxed with a top/bottom defected side. Table 1 summarizes the type and number of defects shown in Figure S1. From this table, we can see that the total number defects in Junct-2, Junct-3, Junct-4, and Junct-5 are 7, 15, 19, and 30, respectively. Figure 2 shows the five hNTJs. The yellow shaded region represents the left/right (6,6) CNT leads, while the middle region is the extended molecule including the BNNT.

**Table 1:**
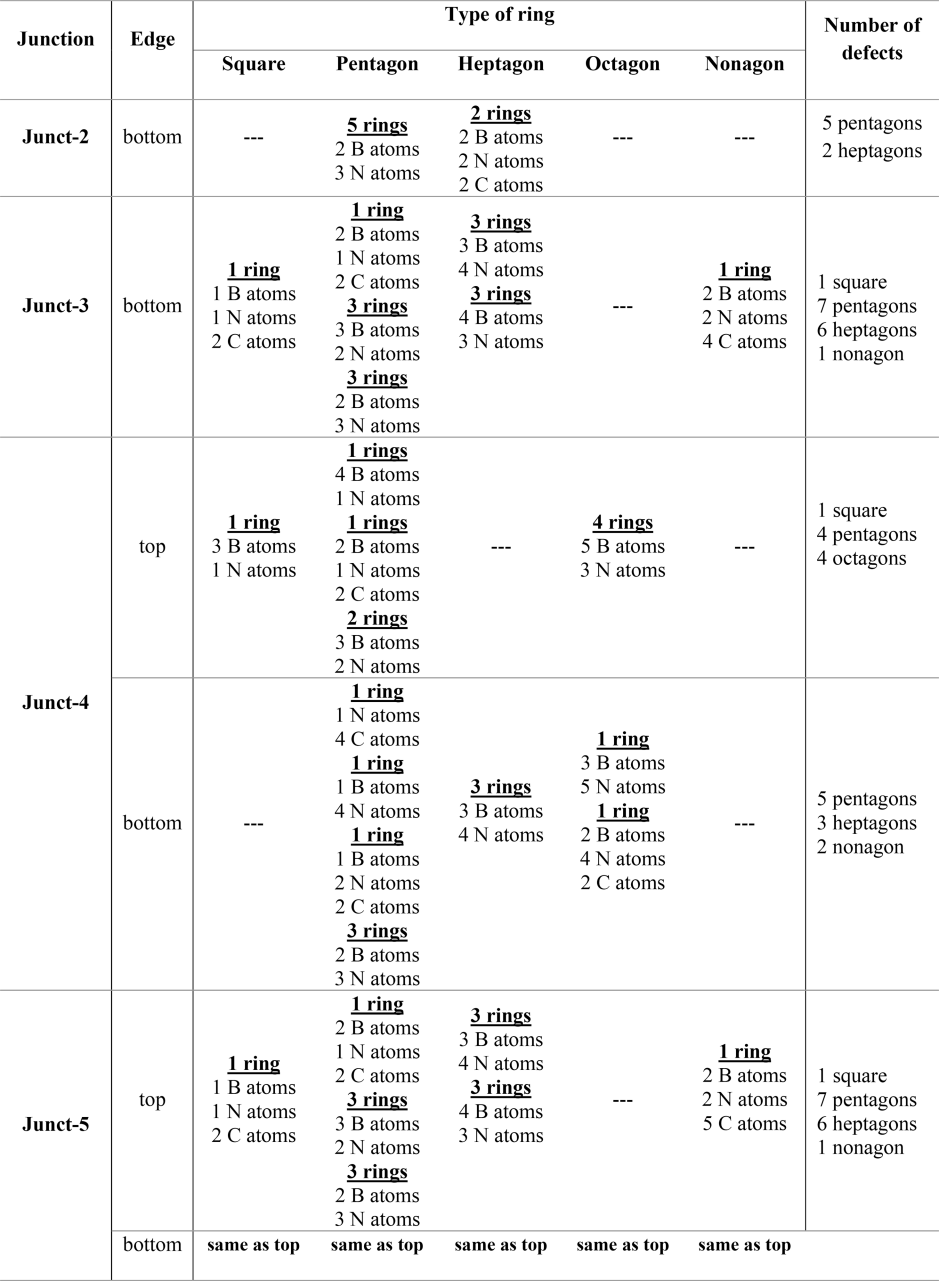
Number and Types of Defects That Emerge in Each of the Relaxed hNTJs

**Figure 2:**
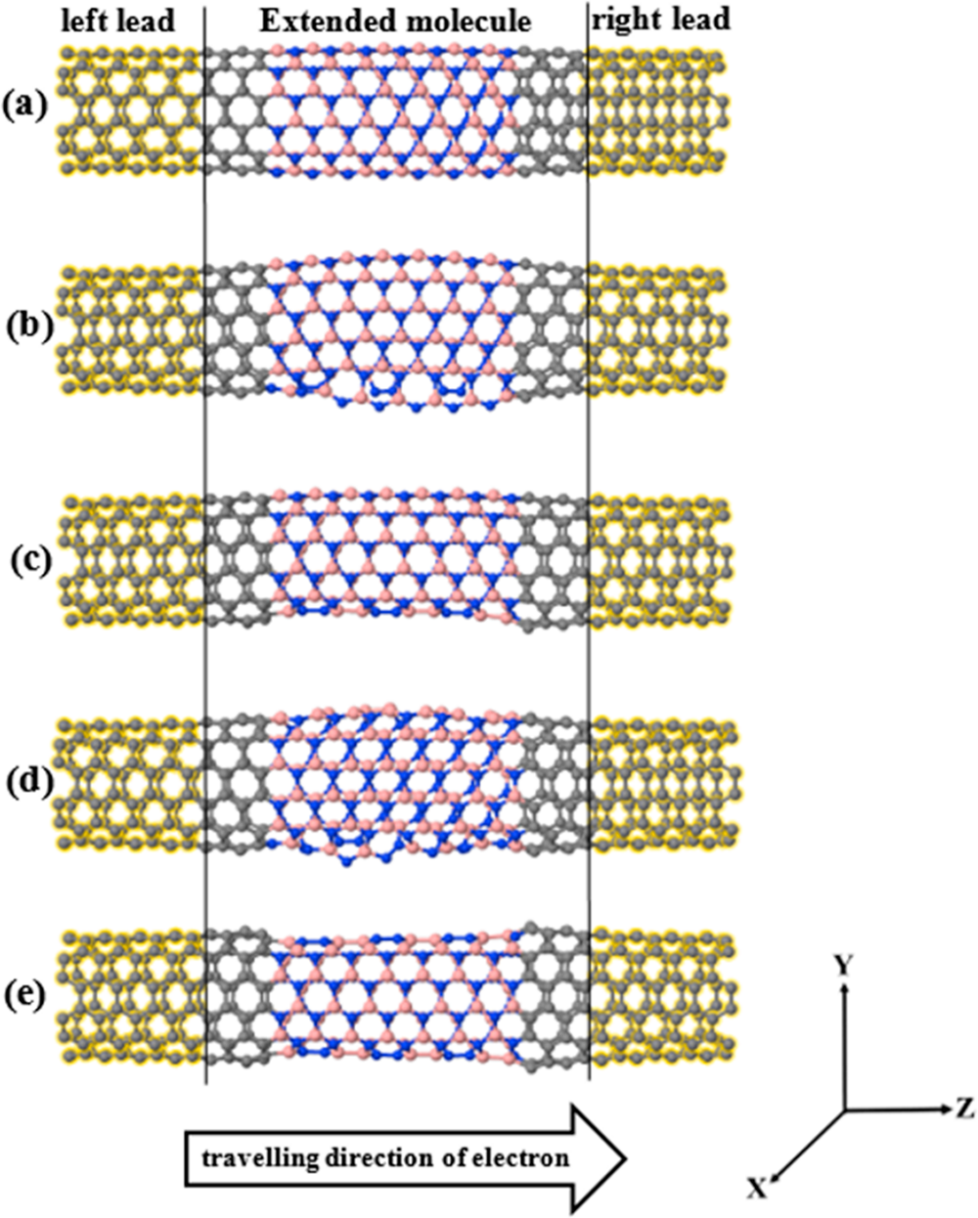
(a) Ideal Junct-1, (b–e) defected Junct-2, Junct-3, Junct-4, and Junct-5, respectively. All junctions are periodic in the *Z*-direction, and finite in the *X* and *Y* directions.

In what follows, we investigate the transmission coefficient *T*(*E*) of the hNTJs shown in Figure 2, which are plotted in Figure 3. The black line in the subfigures of Figure 3 represents the *T*(*E*) from the left lead to the right lead in the absence of the extended molecule (i.e., an ideal (6,6) CNT), which, according to the Landauer description, is equal to the number of open channels (NOC), since these structures (leads) are periodic in the Z direction.^55^ From the same figure we can see that the *T*(*E*) of the hNTJs is obviously inhibited due to the presence of the BNNT. Notice also that Junc-1 is not periodic, since it has the BNNT scatterer, but it is free of defects. However, contrary to what one would expect, the defected hNTJs show in general (specially at the original Fermi level given by DFT) higher conductances than the defect-free Junct-1. This result, which it is a somewhat surprising behavior that ensues from the fact that the defect-free system is highly insulating, can be explained by the increase of electron delocalization generated by the emergence of dangling bonds at the defects (see below).

**Figure 3:**
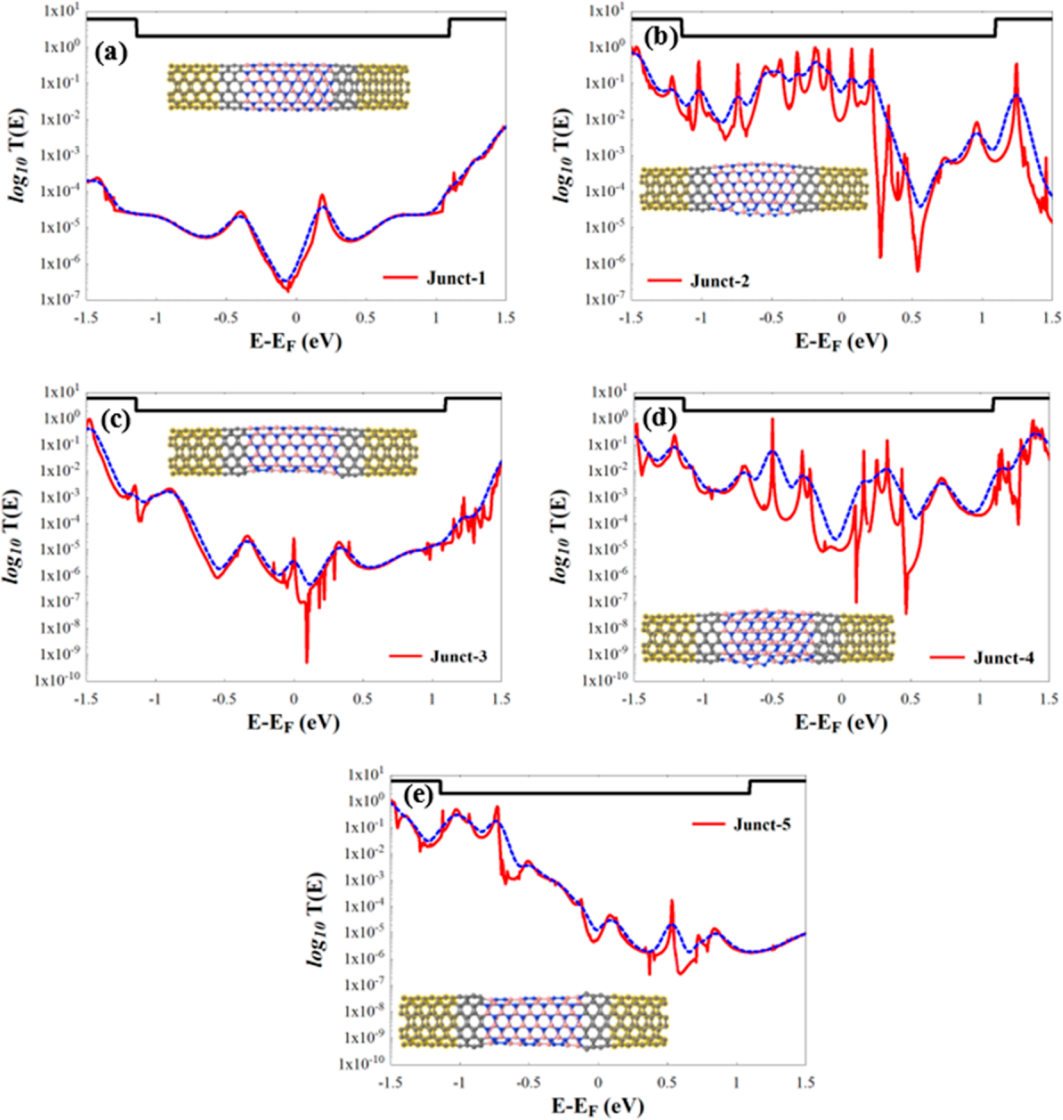
(a–e) *T*(*E*) of the hNTJs shown in Figure 2(a–e), respectively. The dotted blue line in all subfigures shows the conductance at room temperature (*T* = 300 K) and the insets represent the hNTJs. *E*_F_ is the predicted Fermi energy value given by DFT, and it shifted to zero.

From Figure 3, we can also see that there is a relationship between the geometry of hNTJs and the conductance. It is worth mentioning here that in the case of two tubes being linked symmetrically, the formed junction has no bend^77^ as we see in the BNNT region of Junct-1 (Figure 2a), whereas the hNTJs shown in Figure 2(b–e) present curved BNNTs. These curvatures are due to the existence of the defects, which agrees with previous published works.^77−80^ Previous research reported that defects, not only pentagon (five-atom ring) and heptagon (seven-atom ring), lead to bend the nanotubes with different angles, and also tune the electric properties of the nanotubes.^81−86^ Note that sharp bends in CNTs and BNNTs have been observed.^26,80,81,87−89^ BNNTs,^83,90^ which are perfect insulators, might be turned to semiconductors (reduce the energy gap) by bending the tubular structure. Having a look at Figure 1, it is clear that Junct-2 and Junct-4 shown in Figure 1(d,f) have the most curved BNNTs compared to the BNNTs in the rest of hNTJs in Figure 1. These hNTJs (Junct-2 and Junct-4) show the highest conductance as seen in Figure 3(b,d), and this improvement in conductance is not only due to of the existence of the defects, but also due to the curvatures that are created because of the formed defects in the BNNTs regions. Indeed, by assigning topological numbers to local changes in defects with respect to the hexagonal curvature, i.e., pentagons, +1; squares, +2; heptagons, −1; octagons, −2; and nonagons, −3; we can see that the total induced curvatures in each junction are the following: Junct-2, +3; Junct-3, 0; Junct-4, top edge, −2, bottom edge, −4; Junct-5, top and bottom edge, 0. This relates again the enhancement of conductance of Junct-2 and Junct-4 to the curvature. The LDOS calculations in Figure S2(b,d) also show that Junct-2 and Junct-4 have the highest delocalized states around the curvatures and defects, which further confirms the previous claims.

To provide another evidence of the transport enhancement, we calculate the maximum current (*I*) of the hNTJs, shown in Figure 2(a–e) at room temperature for a small finite voltage using the following equation:^91^
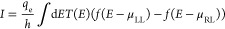
1where *q*_e_ = |*q*_e_| is the electron charge, *h* is Planck’s constant, *T*(*E*) is the electronic transmission coefficient calculated with GOLLUM, *f* is Fermi–Dirac distribution function, *f*(*E* – μ) = 1/(1 + e^(*E−μ*)/K_B_*T*^), with μ_LL_ and μ_RL_ being the electrochemical potentials of the left lead and right lead, respectively, and *T* is the temperature. The resulting currents are shown in Figure 4.

**Figure 4:**
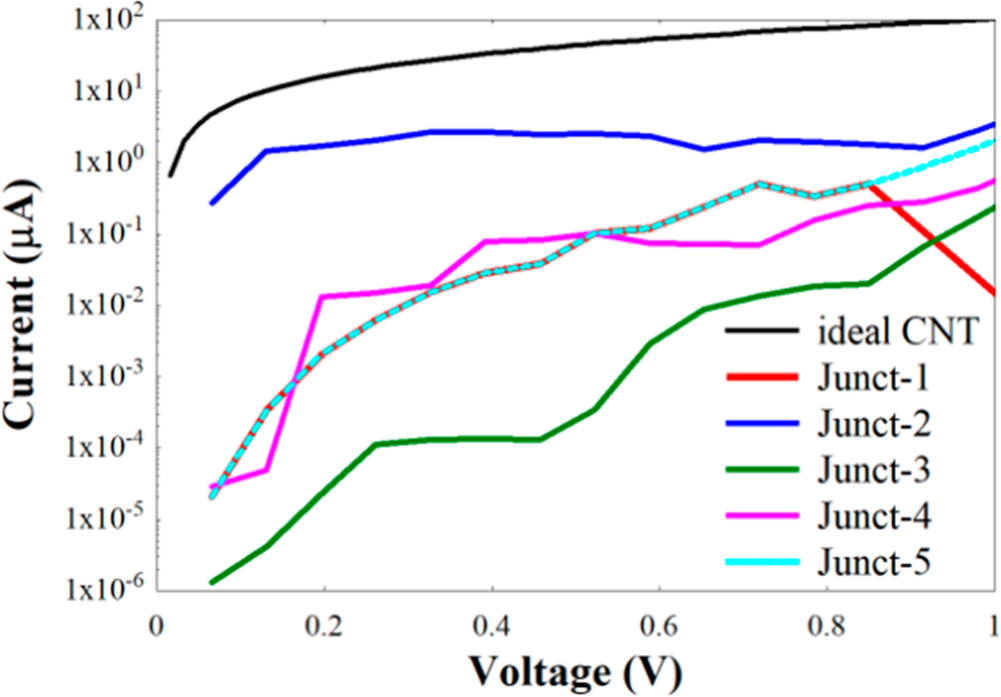
Currents of the hNTJs displayed in Figure 2(a–e).

Once again, Figure 4 shows that there is in general an increase in the current of the hNTJs with more defects shown in Figure 2(b–e), compared to the ideal Junct-1. This increase is due to the presence of defects in the BNNT, which improve the transmission of the junction as compared to that of the insulating BNNT without defects. Notice, however, that the smallest current in most of the bias window is that of Junct-3. The current of Junct-4 can also be smaller than that of Junct-1 in some bias ranges. These two last junctions are characterized by the presence of a somewhat periodic array of defects, which seems to preserve also to a great extent the insulating behavior of the BNNT part. Notice as well that the current of Junct-1 is almost equal to that of Junct-5 in most of the bias range, which is due to the similarity between both junctions, since Junct-5 is equivalent to Junct-1 with two less rows of atoms at the top and bottom edges. Finally, in terms of current, the best junction is Junct-2.

Apart from defects and curvature, another factor that can greatly influence the transport properties is the width of the scattering region. Narrower BN regions can be built again with the sculpturene method, as can be seen in Figure 5. Figure 5a shows the AA-staked zigzag bi-hNRs displayed in Figure 1c (top subfigure) after eliminating a complete row of the boron and nitrogen atoms from the upper edges of the top AA-staked zigzag bi-hNRs and the lower edges of the bottom AA-staked zigzag bi-hNRs. We repeated the same strategy with the initial supercell shown in Figure 5b by removing completely two rows of the boron and nitrogen atoms. Later, we let them relax to form the hNTJs shown in Figure 5(c,d). We call these junctions with narrower BN regions Junct-6 and Junct-7, respectively.

**Figure 5:**
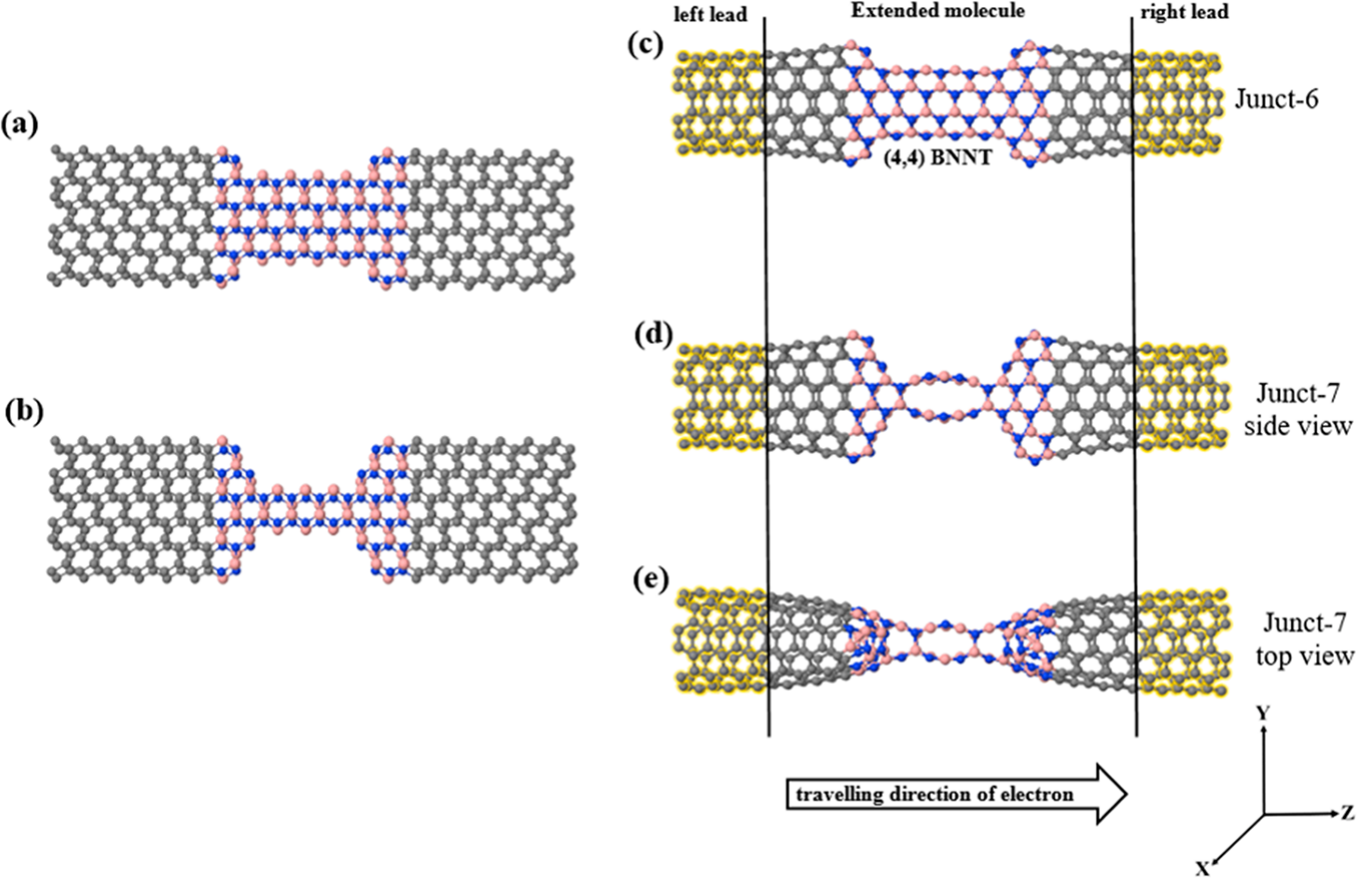
(a,b) Initial zigzag AA-staked bi-hNRs, (c,d) the obtained hNTJs (Junct-6 and Junct-7) after relaxing the initial supercells in (a,b), and (e) top view of Junct-7 shown in (d). The yellow shaded region shown in (c–e) is the (6,6) CNT as left and right leads. All structures are infinite in the *Z* direction and finite in the *X* and *Y* directions.

Figure 5c shows that the middle region (BN) of Figure 5a is finally relaxed into a (4,4) BNNT, whereas the middle BN region of Figure 5b relaxes into a bridge of multichains composed of boron and nitrogen atoms, as can be seen in Figure 5d (side view) and Figure 5e (top view). Figure 6 shows the resulting *T*(*E*) and currents of Junct-6 and Junct-7. As can be seen, there is an extreme drop of the conductance compared to the conductance of the hNTJs shown in Figure 2, i.e., as the width of the BN region is reduced, the conductance of the hNTJs decreases.

**Figure 6:**
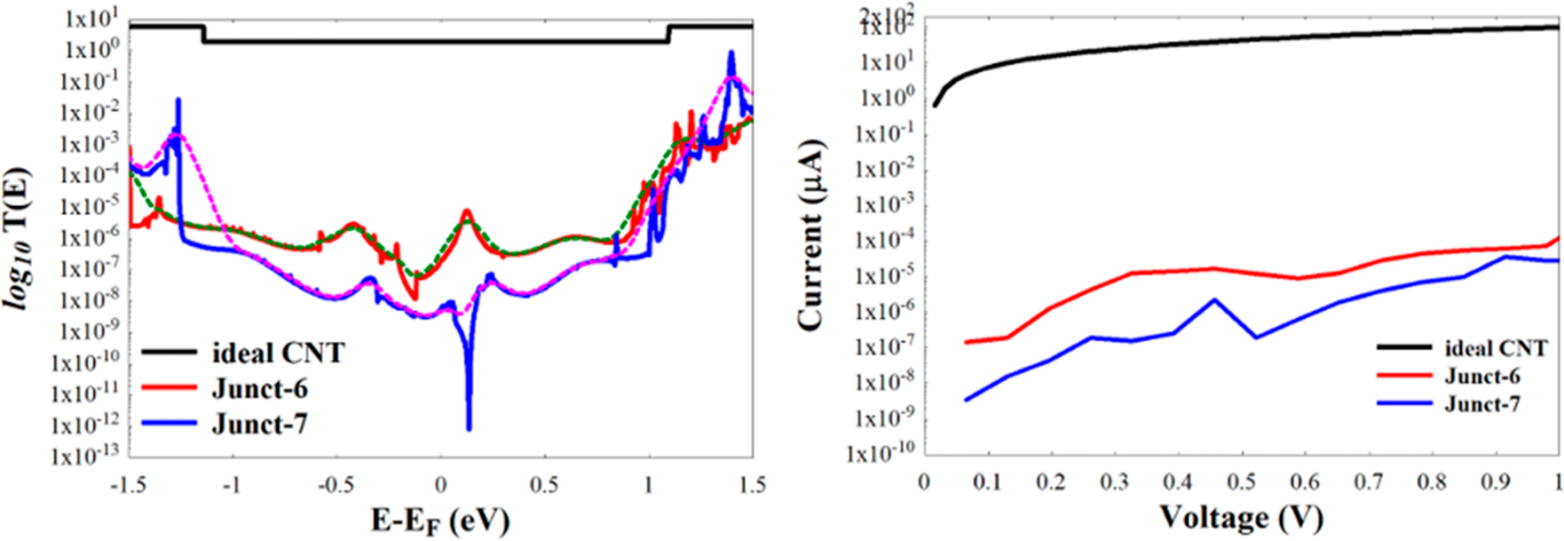
*T*(*E*) (left) and current (right) of Junct-6 and Junct-7 shown in Figure 5(c,d). The dotted lines in the left subfigure shows the conductance calculated at room temperature of Junct-6 and Junct-7.

For a deeper understanding of the evolution of the conductance and current of the heterojunctions shown in Figure 2 and Figure 5, we calculated the Local Density of States (LDOS) in an energy window around *E*_F_, from −0.1 eV to +0.1 eV, as shown in Figure S2. From this figure it is clear that the defects in Junct-2 and Junct-4 give rise to more delocalized states around the *E*_F_, leading to possible paths through which the electrons can travel more easily, whereas the defects in the rest of the heterojunctions (Junct-1, Junct-3, Junct-5, Junct-6, and Junct-7) do not give additional states in the BN part around *E*_F_, keeping the central part roughly insulating. This explains then the enhancement in conductance and current of Junct-2 and Junct-4.

Additionally, we note that resonances that appear around *E*_F_ in *T*(*E*) should lead to enhancement of the Seebeck coefficient (*S*) of the hNTJs. To demonstrate this, we calculate *S* for all hNTJs, shown in Figure S3. The obtained values of *S*, listed in Table S1, are remarkable and illustrate again that hNTJs in contact with ideal CNTs can lead to a rather good thermoelectric performance. More research would be needed in any case to further demonstrate the enhancement in the thermoelectric properties of the hNTJs, which is not the main target of this work.

*Conclusions:* In this work, we have investigated the electronic and transport properties of defects that might be created in hNTJs using the sculpturene method, or manufactured by other methods, and the influence of other factors such as the curvature induced by the defects. Our results show that the transmission from left lead to right lead through the defective BNNTs is significantly reduced compared with that for ideal CNTs. On the other hand, the presence of defects and the induced curvature lead to an enhancement in the conductance of hNTJs, compared to that of the free-defect hNTJ. We also demonstrated that narrowing the BNNT region leads to remarkable decreases of the conductance. Our LDOS analysis explains the high transmission of Junct-2 and Junct-4, which show highly delocalized states around *E*_F_, while for the other hNTJs, shows no evidence of such states in the scattering region. Furthermore, our findings demonstrate that defects give rise to improvements in the Seebeck coefficient (S) of the hNTJs. This work lays then the foundation for the design of nanoscale sensors that, through the change in their transport properties, can act as detectors of particles that generate defects in materials.

## Data Availability

The data that support the findings of this study are available from the corresponding author upon reasonable request.

## Supporting Information Available

Additional analysis and calculations, including a figure with the types of defects and calculations of the local density of states and the Seebeck coefficients (PDF)
Transparent Peer Review report available (PDF)
