# Supplementary material for: Defect-Induced Transport Enhancement in Carbon–Boron Nitride–Carbon Heteronanotube Junctions
Source: J Phys Chem Lett. 2023 Feb 16;14(8):2056–64. doi: 10.1021/acs.jpclett.3c00004 (PMC9986950; doi:10.1021/acs.jpclett.3c00004)
Supplement: Supplementary file 1 — jz3c00004_si_001.pdf [file jz3c00004_si_001.pdf]

Defect-Induced Transport Enhancement in Carbon-Boron Nitride-Carbon Heteronanotube Junctions

Laith A. Algharagholy[1], and V. M. García-Suárez[2*]

[1]Department of Physics, College of Science, University of Sumer, Al Rifaee, Zip: 64005, Thi-Qar, Iraq.

[2]Departamento de Física, Universidad de Oviedo & CINN, Oviedo, 33007, Spain.

*Corresponding author: vm.garcia@cinn.es

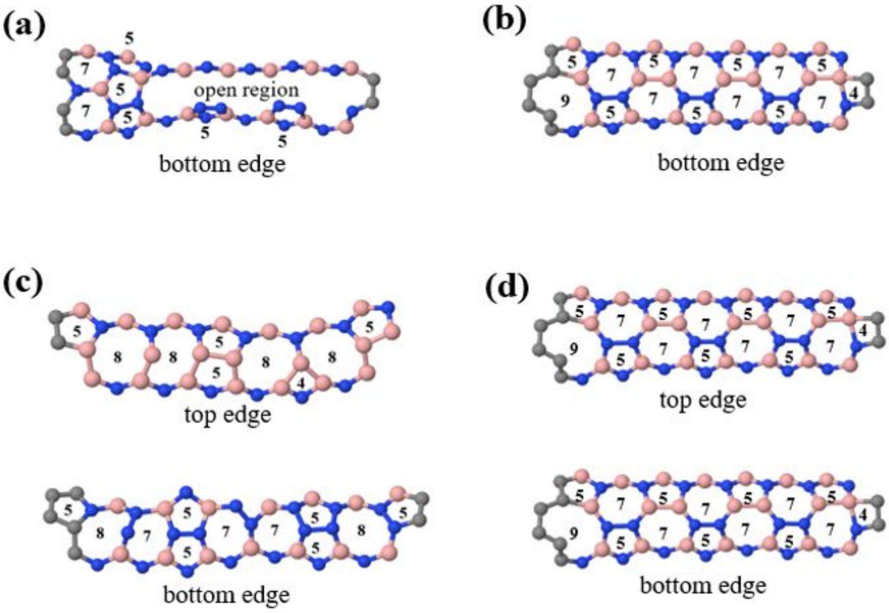

Figure S1: Types of defects of the heterojunctions shown in figure 1 (d-g), **(a)** bottom edge of Junct-2, **(b)** bottom deffected edge of Junct-3, **(c,d)** top and bottom edges of Junct-4 and Junct-5 respectivley. The numbers in subfigures refer to the number of atoms in the rings.

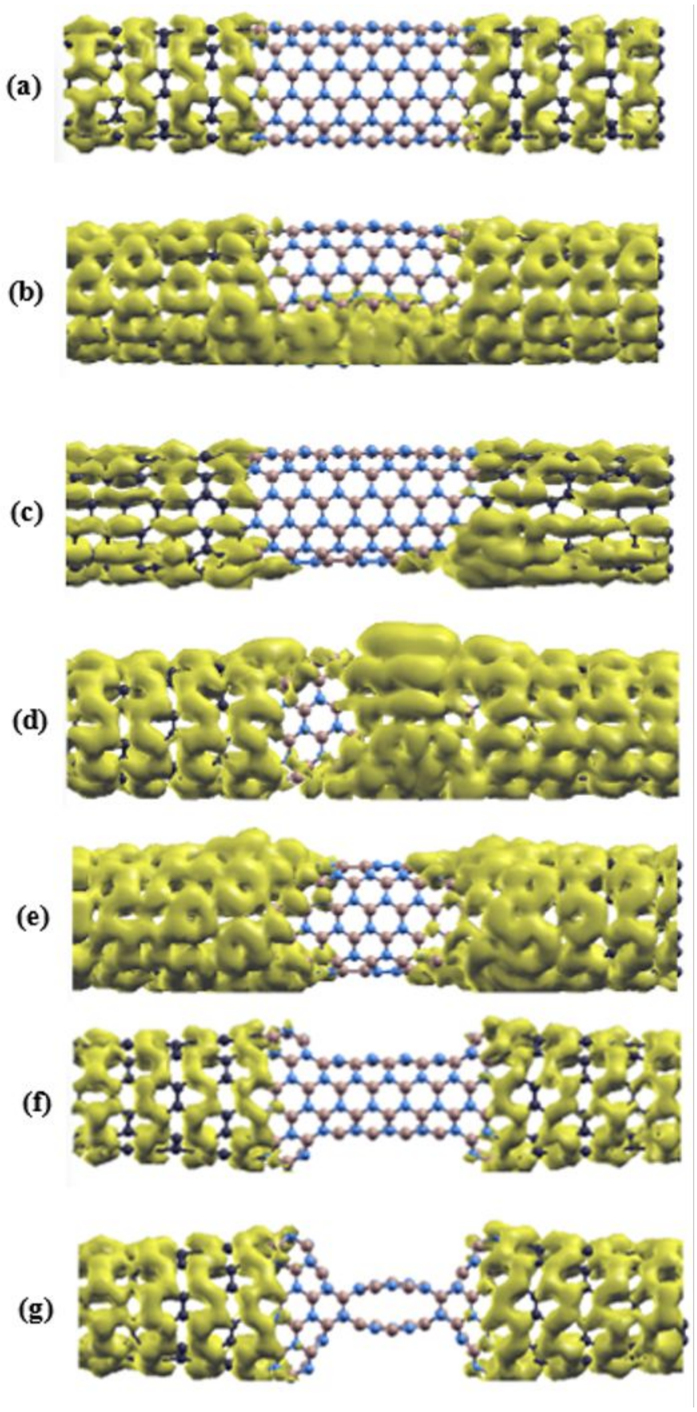

Figure S2: LDOS of the heterojunctions **(a-g)** Junct-1, Junct-2, Junct-3, Junct-4, and Junct-5, Junct-6, and Junct-7 respectively.

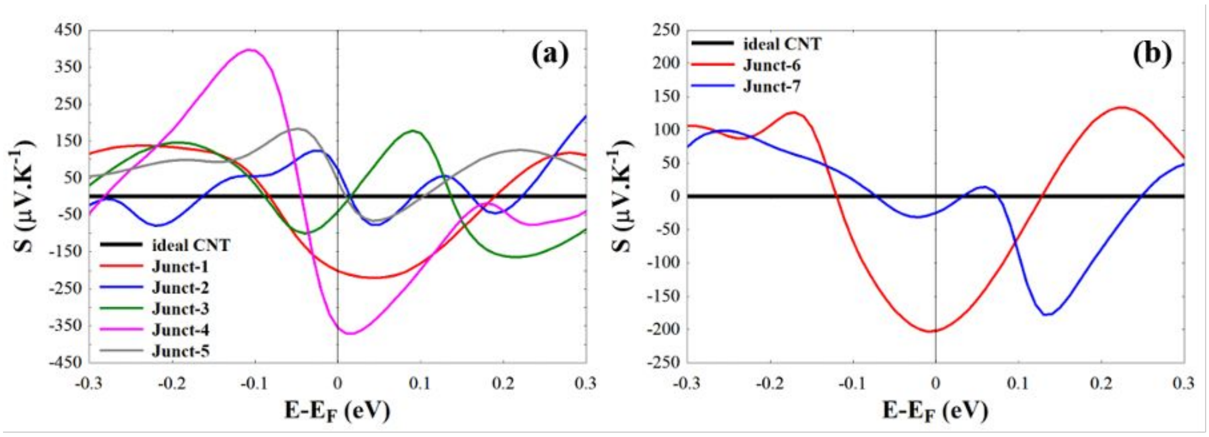

Figure S3: Seebeck coefficient *S* of the hNTJs shown in **(a)** figure 2(a-e), and **(b)** figure 5(c,d).

Table S1: Values of the Seebeck coefficient *S* of the hNTJs shown in figure 2 and figure 5.

hNTJs	S ($\mu V.K^{-1}$)	Type
**Junct-1**	-200.98	n-type
**Junct-2**	72.60	p-type
**Junct-3**	-41.61	n-type
**Junct-4**	**-355.82**	n-type
**Junct-5**	45.52	p-type
**Junct-6**	-201.58	n-type
**Junct-7**	-24.21	n-type
