# Supplementary material for: Defect-Induced Transport Enhancement in Carbon–Boron Nitride–Carbon Heteronanotube Junctions
Source: J Phys Chem Lett. 2023 Feb 16;14(8):2056–64. doi: 10.1021/acs.jpclett.3c00004 (PMC9986950; doi:10.1021/acs.jpclett.3c00004)
Supplement: Supplementary file 2 — jz3c00004_si_002.pdf [file jz3c00004_si_002.pdf]

jz-2023-000046.R1

Name: Peer Review Information for "Defect-Induced Transport Enhancement in Carbon-Boron Nitride-Carbon Heteronanotube Junctions"

First Round of Reviewer Comments

Reviewer: 1

Comments to the Author

The paper deals on how ballistic transport is enhanced in boron nitride nanotube sandwiched between two carbon nanotube leads. The enhanced conductance seems due to defects act as conducting sites within the gap, the ideal BN junction should show no conductance except for the tunnelling between leads. Based on the next concerns I ask for major revision before publication.

List of concerns:

- References are recent papers, but the key references in the past are missing. I wonder untill what point this should be covered just based on the existing review papers.

- Local density approximation (LDA) for the exchange correlation functional gives too much binding...but it is not bad to describe vdw systems. Please add a comment.

- With respect to the initial configurations, I have my doubts that energetically NTs are more stable because the strain energy when forming the nanotubes is a destabilizing factor.

- I am missing an analysis of the curvature with respect to the hexagonal lattice. We can assign local changes in defects with respect to the hexagonal curvature so that pentagons have +1 change in topology, and heptagons -1. Squares +2 and octagons -2 and so on. When counting the number of defects we see that the total induced curvature with respect the perfect hexagonal is given in the following: Junct-2 , +3; Junct-3, 0; Junct-4, top edge =-2, bottom edge = -4; Junct-5, top and bottom edge= 0. Thus, it seems to me that the enhancement in conductance is related to changes in curvature induced by the BN region more than with the width. Note that after looking in detail to the structure of Junct-3 it has a large strain as to say anything even related to curvature.

Author's Response to Peer Review Comments:

Reply to the comments

The paper deals on how ballistic transport is enhanced in boron nitride nanotube sandwiched between two carbon nanotube leads. The enhanced conductance seems due to defects act as conducting sites within the gap, the ideal BN junction should show no conductance except for the tunnelling between leads. Based on the next concerns I ask for major revision before publication.

We thank the Reviewer for carefully reading the manuscript and for valuable comments and suggestions. We have added additional text and modified the article following the suggestions (changes are highlighted in blue) and also shorten the abstract to comply with the Journal guidelines. We reply below to all concerns.

List of concerns:

- *References are recent papers, but the key references in the past are missing. I wonder untill what point this should be covered just based on the existing review papers.*

We have added relevant references and cited them in the introduction section. They are numbered in the new version as 34, 54, 62, 63, and 65.

34 - Charlier, J.-C., T. Ebbesen, and P. Lambin, *Structural and electronic properties of pentagon-heptagon pair defects in carbon nanotubes.* Physical Review B, 1996. **53**(16): p. 11108.

54 - Blase, X., et al., *Structural and electronic properties of composite BxCyNz nanotubes and heterojunctions.* Applied Physics A, 1999. **68**: p. 293-300.

62 - Stephan, O., et al., *Doping graphitic and carbon nanotube structures with boron and nitrogen.* Science, 1994. **266**(5191): p. 1683-1685.

63 - Redlich, P., et al., *B C N nanotubes and boron doping of carbon nanotubes.* Chemical physics letters, 1996. **260**(3-4): p. 465-470.

65 - Blase, X., et al., *Theory of composite B x C y N z nanotube heterojunctions.* Applied Physics Letters, 1997. **70**(2): p. 197-199.

- *Local density approximation (LDA) for the exchange correlation functional gives too much binding...but it is not bad to describe vdw systems. Please add a comment.*

We have added the following comment and reference in the article (page 3):

“Notice that the LDA usually overestimates the binding, giving distances between atoms a bit smaller than the real ones, but works properly in general to describe van der Waals interactions [75] such as those present in the initial stages of the design of the sculpturenes.”

- *With respect to the initial configurations, I have my doubts that energetically NTs are more stable because the strain energy when forming the nanotubes is a destabilizing factor.*

We agree with the Reviewer that the strain energy is a destabilizing factor that affects the formation of the nanotubes in the initial stages. Indeed, this was a concern when the sculpturene method was developed, as evidenced by the usually large forces and stresses present in the initial stages. However, by carefully choosing the initial configurations (both layers close enough), the process often leads to a nanotube after several relaxation steps. In addition, in some cases used in other studies, the process can be speeded up by using molecular dynamics (this was not needed in the present study). We have commented this in the article as follows:

“Notice that, even though the strain can be large and act as a destabilizing factor in the initial stages of the formation of the nanotubes, the structures naturally evolve to such configurations after several relaxation steps, provided the initial ribbons are close enough to each other.”

- *I am missing an analysis of the curvature with respect to the hexagonal lattice. We can assign local changes in defects with respect to the hexagonal curvature so that pentagons have +1 change in topology, and heptagons -1. Squares +2 and octagons -2 and so on. When counting the number of defects we see that the total induced curvature with respect the perfect hexagonal is given in the following: Junct-2 , +3; Junct-3, 0; Junct-4, top edge =-2, bottom edge = -4; Junct-5, top and bottom edge= 0. Thus, it seems to me that the enhancement in conductance is related to changes in curvature induced by the BN region more than with the width. Note that after looking in detail to the structure of Junct-3 it has a large strain as to say anything even related to curvature.*

We thank the Reviewer for raising this question and for the analysis of the topology of the defects. Indeed, the analysis of the effect of the curvature was missing in the article. We have modified the results section of the article and added also the following paragraph to comment on this (page 9):

“From figure 3, we can also see that there is a relationship between the geometry of hNTJs and the conductance. It is worth mentioning here that in case of two tubes are linked symmetrically, the formed junction is with no bend 77 as we see in BNNT region of Junct-1 (figure 2a), whereas the hNTJs shown in figure 2(b-e) present curved BNNTs, these curvatures are due to the existing of the defects, which agree with previous published works77-80. They reported that defects, not only pentagon (five-atom ring) and heptagon (seven-atom ring), lead to bend

the nanotubes with different angles, and also tuning the electric properties of the nanotubes81-86, note that the sharp bends CNTs and BNNTs have been observed26,80,81,87-89. BNNTs83,90, which are perfect insulators, might be turn to semiconductors (reduce the energy gap) by bending the tubular structure. Having a look at the figure 1, it is clear that Junct-2 and Junct-4 shown in figure 1(d,f) have the most curved BNNTs compared to the BNNTs in the rest of hNTJs in figure 1. These hNTJs (Junct-2 and Junct-4) show the highest conductance as seen in figure 3(b,d), and this improvement in conductance is not only due to of the existence of the defects, but also due to the curvatures that are created because of the formed defects in the BNNTs regions of the hNTJs. Indeed, by assigning topological numbers to local changes in defects with respect to the hexagonal curvature, i.e. pentagons +1, squares +2, heptagons -1, octagons -2 and nonagons -3, we can see that the total induced curvatures in each junction are the following: Junct-2 , +3; Junct-3, 0; Junct-4, top edge, -2, bottom edge, -4; Junct-5, top and bottom edge, 0. This relates again the enhancement of conductance of Junct-2 and Junct-4 to the curvature. The LDOS calculations in figure S2(b,d) also show that Junct-2 and Junct-4 have the highest delocalised states around the curvatures and defects, which further confirms the previous claims."
